# Interaction among participants in a collective intelligence experiment: an emotional approach

**DOI:** 10.3389/fpsyg.2024.1383134

**Published:** 2024-05-15

**Authors:** Santos Orejudo, Raquel Lozano-Blasco, Pablo Bautista, Montserrat Aiger

**Affiliations:** ^1^Department of Psychology and Sociology, University of Zaragoza, Zaragoza, Spain; ^2^Department of Educational Sciences, University of Zaragoza, Zaragoza, Spain

**Keywords:** collective intelligence, collective learning, sentiment analysis, cyberbullying, platform, online experiment

## Abstract

**Introduction:**

The construct of collective intelligence assumes that groups have a better capacity than individuals to deal with complex, poorly defined problems. The digital domain allows us to analyze this premise under circumstances different from those in the physical environment: we can gather an elevated number of participants and generate a large quantity of data.

**Methods:**

This study adopted an emotional perspective to analyze the interactions among 794 adolescents dealing with a sexting case on an online interaction platform designed to generate group answers resulting from a certain degree of achieved consensus.

**Results:**

Our results show that emotional responses evolve over time in several phases of interaction. From the onset, the emotional dimension predicts how individual responses will evolve, particularly in the final consensus phase.

**Discussion:**

Responses gradually become more emotionally complex; participants tend to identify themselves with the victim in the test case while increasingly rejecting the aggressors.

## 1 Introduction

The construct of collective intelligence has become increasingly popular in a variety of scientific domains, as it provides a theoretical and empirical framework that allows researchers to analyze how groups can perform better than individuals. Many studies have been conducted in a small-group environment where participants collectively generate a product that researchers can compare with an equivalent product resulting from individual actions or answers. Sharing a common methodology, the studies by Woolley et al. (2010), Engel et al. (2014), Meslec et al. (2016), Aggarwal and Woolley (2018), Aggarwal et al. (2019), Woolley and Aggarwal (2020) are a good example of the practical application of the premise of collective intelligence. Although those studies have nevertheless received a certain amount of criticism, researchers who have applied other methods have reached similar conclusions (Navajas et al., 2018; Toyokawa et al., 2019; Pescetelli et al., 2021).

This paradigm of dealing with collaborative group tasks in small groups featuring face-to-face interaction is fundamentally transformed when interactions are transferred to the Internet environment: a “digital space,” as designated by Pescetelli et al. (2021). One of the most important differences between face-to-face and online interaction is that the online environment allows researchers to considerably improve the number of individuals participating and collaborating in tasks. They can thus ascertain whether a large number of jointly interacting people might improve the chance of resolving the open, complex problems that confront humanity today (Lorenz et al., 2011; Hamada et al., 2020). The crowd intelligence construct provides us with a theoretical and empirical framework to approach such problem-solving processes on a collaborative basis in large groups; it is assumed, however, that the participants do not necessarily need to interact with one another to reach the solution (Hamada et al., 2020). One of the major research interests in this field is to ascertain under which conditions group performance can be optimized (Becker et al., 2017; Yahosseini and Moussaïd, 2020; Pescetelli et al., 2021). Key issues in such empirical analyses of the productions of large groups include: (1) ascertaining which is the most adequate method to aggregate potential solutions with the ultimate purpose of confirming that the collective product is better than the individual solution, and (2) studying how such products can be operationalized and quantified. One frequent solution is to devise estimation tasks that are easy to quantify; such tasks, however, seem to be far removed from the more complex, open kind of tasks with which human beings are usually faced in natural settings (Geifman and Raban, 2015).

Another key issue in the study of collaborative tasks is establishing a series of conditions that allow participants to interact with one another to achieve the best solution to a problem. Indeed, if overall performance needs to be improved, such collaborative interaction is a necessary condition (Gürçay et al., 2015; Navajas et al., 2018; Toyokawa and Gaissmaier, 2022), but it also serves as a differentiating factor among diverse types of collective intelligence processes (Hamada et al., 2020). As mentioned above, face-to-face interactions are limited to a reduced number of people, usually four to six individuals; in such contexts, the ideal number of participants in a group is five (Navajas et al., 2018). The number can be increased in some instances, as in particularly demanding tasks that require a more enhanced degree of coordination among members and a more significant amount of incentive (Mao et al., 2016). In the online environment, however, interactive spaces can gather elevated quantities of individuals who can interact with one another to analyze a problem or simply share their experiences regarding a particular product. Their outlook evolves naturally through interactions in a dynamic system (Almaatouq et al., 2020). The task of analyzing and understanding what goes on in such “digital spaces” is thus more complex.

This context thus poses two empirical questions. How does large-group interaction take place in such digital contexts? Is it truly possible to achieve quality solutions to particular problems via online interactions? Both of these issues ultimately lead us to analyze the nature of such interactions (Navajas et al., 2018; Massari et al., 2019) along with further key issues, including social influence processes (Gürçay et al., 2015; Becker et al., 2017; Toyokawa et al., 2019) and the participants’ level of involvement (Hamada et al., 2020). It is also important to define and establish the type of problem/task to be solved (Geifman and Raban, 2015; Sulik et al., 2022). An additional element worthy of study resides in the design of the task itself: the key issue is to ascertain whether the system foresees for the group to produce a consensual solution to the problem or whether the solution should be generated *a posteriori* through aggregation mechanisms that are external to the group (Yahosseini and Moussaïd, 2020).

A considerable number of complex issues are thus involved. Our study aims to analyze the interactions that arise when a large number of people (in our case, close to 800 individuals) jointly explore a task in an interactive environment on an online platform until they propose solutions. One key issue lies in resolving how the productions of a significant number of people should be quantified or measured, since their interactions can only be quantified as a series of thousands of individual events. We thus propose to study how those interactions evolve in the context of a large group that is dealing with an ethical problem consisting in the analysis of a case of cyberbullying. We chose and designed this case with the purpose of encouraging engagement on the part of the participants, who were asked to emit judgments about “what happened.” We propose to study the situation using big data tools designed to analyze emotions (Aggarwal, 2022; Trisna and Jie, 2022; Chan et al., 2023).

It is also essential to grasp the nature of the interactive environment in which a task is carried out. The IT environments in which large group interactions take place can have many different characteristics and goals, ranging from social media (on which information can be shared) to collaborative environments such as Wikipedia (Holtz et al., 2018). Certain tools are specifically designed to create collaborative environments in which information can be shared, joint solutions can be reached, and consensus mechanisms can be established (Geifman and Raban, 2015). This is also the case on the *Collective Intelligence* platform we used in this study: its characteristics are described further below and can also be consulted in Orejudo et al. (2022).

This study’s objective was to analyze how information spreads in an online interaction environment designed according to the principles of collective intelligence. We hypothesize that the emotional dimension plays a key role in this process and predicts how information spreads within that environment.

### 1.1 Collective intelligence

In the field of social behavior, the construct of Collective Intelligence (CI) has become increasingly popular, starting with the study by Woolley et al. (2010) published in the journal Science. The underlying supposition in collective intelligence is that when people work together as a group (intragroup level) on tasks of various kinds, the group’s level of performance is superior to the mean of the performances of its individuals (intrapersonal) on the same tasks. One of the most interesting results of this study is the idea of the “c” factor. Based on the traditional idea of the g-factor of intelligence, the researchers found that the factor analysis carried out on the performance of the groups on the different tasks suggested a single factor that explained most of the variance in the results, the so-called “c” factor. A series of studies by that research team have confirmed that results achieved in face-to-face contexts and in online environments are similar (Engel et al., 2014; Meslec et al., 2016). Other investigations have been conducted featuring tasks of dissimilar nature (Woolley and Aggarwal, 2020); further studies have sought to ascertain what kind of conditions exert a significant influence on results.

Those studies were criticized by other authors (Starkey, 2012; Rowe et al., 2021). After conducting a meta-analysis of CI studies based on the Woolley paradigm, Rowe et al. (2021) called certain results into question, particularly those related to what is known as the c-factor. They pointed out that the level of individual or group performance on tasks can be highly determined by contextual factors and by the nature of the task itself: this, in turn, would limit the validity of the c-factor. In order to take the importance of contextual factors into account, it is necessary to describe them more closely. Woolley et al. (2015) provided an important contribution by proposing two analysis levels: top-down factors (those related to the participants in the task) and bottom-up factors (those related to the conditions in which the interaction takes place). Among personal or top-down factors, the first studies focused on the sex variable, ascribing a greater degree of emotional competency to women. For a group to function well, emotional competency is key: it improves the group’s cohesion and plays a pivotal role in conflict management situations (Curşeu et al., 2015). Other authors have added further considerations related to emotional intelligence (Hjertø and Paulsen, 2016), social sensitivity, and cognitive diversity (Woolley et al., 2010; Aggarwal et al., 2019). Independent replications of the Woolley studies have extended our knowledge regarding these variables: not all results could be replicated. For instance, Bates and Gupta (2017) ascribed greater importance to the individual characteristics of a group’s members than to the group’s heterogeneity *per se*.

Among possible bottom-up factors, interaction among group members is regarded as a necessary condition for collective intelligence to emerge (De Vincenzo et al., 2017; Bernstein et al., 2018; Dai et al., 2020). Indeed, within the paradigm established by Woolley et al. (2010), the condition of interaction is essential, as the final product is achieved by a group. The level of participation of group members is also relevant. Woolley and Aggarwal (2020) found that groups where turn-taking was more varied tended to perform better, whereas performance was inferior in groups where only certain participants monopolized conversational turn-taking. Bernstein et al. (2018) found that a group’s creativity and the quality of its responses to a previously poorly defined task tended to improve considerably when intermittent interactions were introduced within the group. In their analysis of decision-making contexts, De Vincenzo et al. (2017) found that intermediate transitions were the ones most prone to improve a group’s overall performance. In tasks of an individual nature tackled by a group, Navajas et al. (2018) confirmed that the estimations emitted by participants in the group are better than those emitted by individuals: even one sole previous interaction in the group suffices to improve performance with regard to similar tasks carried out on an individual level.

The interactions within the group emerge from a learning process that is essentially social in nature (Woolley and Aggarwal, 2020). Navajas et al. (2018) found that this specifically occurs when small groups interact with one another, share opinions, contrast differing points of view, and reach agreements. However, other social factors can impact behavior within the group. For instance, factors associated with social prestige can lead to extreme behaviors or can lead the other members to participate less: this is the so-called herd effect, which is more likely to occur in large groups (Fontanari, 2018; Toyokawa et al., 2019; Yahosseini and Moussaïd, 2020; Sulik et al., 2022). Further factors, such as leadership or physical proximity, can act as key elements of social influence (Kabo, 2018). The relevance of social influence has been experimentally proven. For example, the study by Salganik et al. (2006) showed that social influence can determine purchase habits to a greater degree than the quality of the product.

Another characteristic of large groups is the wide diversity of responses generated through interaction: this becomes an essential element of the process. Response diversity is the necessary condition for creative solutions to appear; at the same time, however, it can represent a risk in the attempt to reach a consensus (Klieger, 2016; Mann and Helbing, 2017; Yahosseini and Moussaïd, 2020; Pescetelli et al., 2021; Sulik et al., 2022). One of the most relevant issues is ascertaining under which conditions such response diversity can become the basis for the emergence of collective intelligence (Sulik et al., 2022). On the one hand, we need to grasp how such diversity emerges. On the other hand, it is necessary to determine what kind of conditions can work in favor of achieving consensus. Thus, in the first case, the herd effect, or, on the other hand, a low degree of participation on the part of certain group members, can be two of the factors that would hinder response diversity. Toyokawa et al. (2019) suggest that the nature of the task can also impact the diversity of responses: more open tasks lead to a broader variety of responses, while more closed tasks lead to a more reduced variety. On the other hand, certain authors propose that a productive way of taking advantage of diversity is to have an agent working inside the system who plays the role of “facilitator,” i.e., who controls and orients the group in its realization of the task (Bigham et al., 2018). In digital environments, the facilitator task is delegated to an automatic system (Gimpel et al., 2020). Such systems are difficult to design. The social prestige of responses is one of the elements that work most in favor of consensus. Still, nothing guarantees that responses that enjoy considerable social prestige are necessarily the best (Lorenz et al., 2011). An attempt to improve this system would consist in ensuring a genuinely collective generation and distribution of responses while optimizing the participation of all agents involved in the task. This approach can be carried out in real-time, as was the case in the system applied in the current study (Orejudo et al., 2022).

### 1.2 *Collective learning:* a collective intelligence platform

As mentioned above, this study belongs to a series of studies on the subject of collective intelligence in online environments. Such environments allow researchers to generate a series of interactions within large groups of people. However, the systems employed can be thoroughly different, and their design determines how the interaction is produced. The Collective Intelligence platform^1^ on which our study was carried out has been designed and developed by researchers. Its goal is to generate an interaction model that allows for the emergence of high-quality solutions to the tasks in question (Orejudo et al., 2022). To achieve this, the platform is designed to seek to address some of the problems that emerge in large-group interactions: for example, the emergence of extreme responses, the copying of responses emitted by prestigious members of the group, the proliferation of multiple solutions, and even the fact that certain participants provide no response whatsoever. The system divides the procedure into successive work phases that can be grouped into three main blocks: an individual work phase (Phase 1), several small-group work phases (Phases 2, 3, 4, and 5), and two final phases oriented toward establishing consensus (Phases 6 and 7).

In the first phase, each participant works alone on the task (Phase 1). Once this has occurred, the system generates interactive dynamics among the participants, exposing each one of them to the information/responses generated by four “neighbors.” After viewing the four neighbors’ answers, the user can modify his/her response or choose to copy one of his/her neighbors’ responses. This all occurs in Phases 2, 3, 4, and 5. All phases are anonymous, thereby preventing that the prestige enjoyed by certain participants might condition the interaction (Bernstein et al., 2018). Using the possibilities made available by computation, each phase presents the user with a different set of “neighbors,” thereby ensuring that information is distributed throughout the entire network. This successive set of phases in small groups has the purpose of generating interactions with a manageable number of neighbors to ensure that participants do not suffer from information overload (Garg et al., 2022); it is also designed for information to be distributed all across the network by means of neighbor rotation in each phase. An environment is thus achieved with a number of participants that guarantees good solutions: five (Navajas et al., 2018; Toyokawa et al., 2019); moreover, the information can be spread across the network in a broader dispersion than would be achieved in small groups, thus avoiding another limitation associated with face-to-face interactions: turn-taking, i.e., when turns are monopolized by a limited number of participants (Mann and Helbing, 2017).

Phases 6 and 7 have the goal of achieving consensus and allowing participants to take the popularity of responses into account as an additional mechanism that enables them to evaluate their own contributions. The system generates a response popularity indicator designed to resemble the prestige effect: in this case, the “prestige” factor emerges through participants’ tendential preference for certain responses during the entire interactive process (Phases 2, 3, 4, and 5). The function of this indicator is to provide participants with feedback as a basis for a consent mechanism that will lead to a set of adequate solutions ranked by participants, while avoiding the factor of group member prestige that would have conditioned their participation and choices. This mechanism of facilitation (Lorenz et al., 2011) modulates the heterogeneity of responses by applying a popularity system. In the final two phases of interaction, once the responses have circulated through the network and have been evaluated by participants, a progressive procedure of response selection emerges, eliminating less frequent responses and exposing participants to the most popular ones. The basic supposition is that after that interaction procedure, the most popular responses in the network will be of high quality (Orejudo et al., 2022). That pruning method thus acts as a virtual moderator of the entire group, transferring a portion of the process of the previously described moderator role to AI (Bigham et al., 2018).

The system is flexibly designed, with the purpose of allowing researchers to previously establish the duration of each of the seven phases of each work session, the percentage of interchanges that take place in the group phases (Phases 2 to 5), and the number of response eliminations that take place in the consensus phases (Phases 6 and 7). Moreover, the system collects and stores all participant interventions each time they elaborate or copy an idea and introduce it into the system by choosing the “Save Answer” option. The resulting data generated by the platform can thus be analyzed, identifying which participant made the contribution and at which moment in time.

### 1.3 Sentiment analysis. A natural-language-based proposal for analyzing productions

One of the situations we encounter in studies of collective intelligence (CI) is the emergence of a large quantity of responses, particularly when the number of participants is elevated, and the task is open (Toyokawa et al., 2019). This leads to a methodological difficulty in analyzing the interaction dynamics generated by participants in a CI process, even when the task is designed to produce consensus (Orejudo et al., 2022). Sentiment analysis (Lozano-Blasco et al., 2021) can offer a valid alternative if we want to analyze the emotional dimension of a text.

Sentiment analysis is a procedure that allows us to understand people’s opinions or attitudes regarding a situation, a product, a service, or a concept, as expressed in a written text (Ceron et al., 2013; Yu et al., 2013). Sentiment analysis initially emerged in a business context, with the goal of finding hidden information in user comments that served as a basis for their business strategies and decisions (Aggarwal, 2022). In other words, sentiment analysis is a social thermometer that measures the feelings/emotions of users, uncovering tendencies in their opinions and comments on social networks (Chan et al., 2023). Specialized software is available for the procedure (Fang et al., 2018).

Sentiment analysis is capable of analyzing a text – a tweet, a post, or a video – in order to determine whether it transmits a positive, negative, or neutral feeling (Aggarwal, 2022). Certain specific tools allow the researcher to determine further variables, including the emotional diversity of a text, its objectivity, or its degree of irony (Trisna and Jie, 2022). It is even possible to elicit which are the most predominant emotions in a text, as shown in Plutbick’s theory (Plutchik, 2001; Trisna and Jie, 2022). Sentiment analysis can likewise identify subjects and themes by grouping them into clusters.

These software products are based on natural language processing (NLP) (Fang et al., 2018). Although such categorization can be done manually, the most common current approach is the application of automated computational methods that are not externally supervised, such as Google Cloud Natural Language API and IBM Watson Natural Language Understanding (Hu and Liu, 2004; Yu et al., 2013). Two types of sentiment analysis can be distinguished: (a) analysis based on the wordlists that associate specific words with a certain feeling or emotion, and (b) analysis based on automatic learning using algorithms that learn to identify feelings (Jiang et al., 2011; Syahrul and Dwi, 2017). As a final result, each text receives a numerical value associated with a quantitative value, which can be emotional polarity or the degree of subjectivity (Jiang et al., 2011; Syahrul and Dwi, 2017).

Regardless of the chosen method, the analysis of interaction always represents a considerable challenge (Evans et al., 2011). When the task is of an emotional nature, researchers have the viable option of applying means of analyzing large quantities of data through emotional evaluation. In this study, we propose to use sentiment analysis to analyze moral judgments emitted about a prototypical case of cyberbullying.

### 1.4 Cyberbullying from an emotional basis

Cyberbullying is a bullying subtype that is carried out in an online environment and fulfills the characteristics of intentionality, abuse of power, and reiteration (Patchin and Hinduja, 2006; Smith et al., 2008). However, the complexity of identifying these characteristics in online environments (which are in constant evolution, and which presuppose the perpetrator’s connection to a device with an Internet connection) implies that this phenomenon is different from mere aggression (Patchin and Hinduja, 2015). Among the multiple typologies of cyberbullying, we find one that is particularly frequent among adolescents: sexting, a type of cyberbullying focused on spreading images or videos with sexual content on the Internet without the owner’s consent (Lounsbury et al., 2011). The prevalence of sexting in adolescence is around 36% (Gámez-Guadix et al., 2017).

Scientific research highlights the relevance of emotional responses to cyberbullying in both aggressors and victims (Hemphill et al., 2015; Arató et al., 2020; Lo Cricchio et al., 2021; Jiang et al., 2022). Emotional competencies have been identified as protective factors against cyberbullying (Guerra et al., 2021). Guerra et al. (2021) and Jiang et al. (2022) associate low levels of emotional competency and emotional control with the realization of acts of cyberaggression (Guerra et al., 2019). The emotions provoked in victims of cyberbullying have been well-identified: anger, fear, sadness, and stress (Hemphill et al., 2015).

The perpetuation of cyberbullying is also associated with low levels of empathetic responsibility (Arató et al., 2020) and a high degree of moral disconnect from the perpetrated act (Paciello et al., 2020; Lo Cricchio et al., 2021). The cyberbully uses such mechanisms to inhibit feelings of remorse (Wang et al., 2016) or pain in the face of the victim’s suffering (Leduc et al., 2018). Inversely, low levels of moral disconnect and high levels of empathetic responsibility are directly linked with non-implication in acts of cyberbullying since people with such levels understand the harm that can be done to other human beings (Perren and Gutzwiller-Helfenfinger, 2012; Wang et al., 2016; Arató et al., 2020; Paciello et al., 2020).

Emotional response also plays a role in the spectators, i.e., those who observe the act of cyberaggression and can either defend the victim, attack them, or maintain a neutral position (Machackova and Pfetsch, 2016). Spectators who support the aggressor are normalizing their opinions about cyberbullying: they are upholding the appearance of such behavior on the Internet (Machackova and Pfetsch, 2016). Conversely, those spectators who defend the victim are showing high levels of affective and cognitive empathy with them (Machackova and Pfetsch, 2016). Nevertheless, the issue of whether someone tends to support the aggressor, or the victim is apparently more closely associated with temporal, technical, or psychological proximity with the implied person: for instance, a social connection with either the victim, the aggressor, or the other spectators (Pfetsch, 2016). In line with the latter author, it is worthwhile to point out that exposure to acts of cyberaggression leads to greater levels of stress and negative emotions than prosocial or neutral interactions among peers (Caravita et al., 2016). The role of the spectator is key in our study’s approach since, in our case, the participants who are analyzing the case of cyberbullying have not directly taken part in it. However, they are associated with it in their position as spectators since their responses to the task help us to determine whether their emotional implication lies with the victim or with the aggressors.

## 2 Method

### 2.1 Goals and hypothesis

This study thus pursues the goal of verifying whether the emotional dimension of participants’ responses when faced with an ethical task on the platform *Collective Learning* (see Footnote 1) tends to vary in function of the phase in which the interaction takes place. Sentiment analysis via automated software is a method that has been successfully applied in other studies that analyzed interactions in virtual interactive environments where an elevated number of participants emit a large number of responses (Lozano-Blasco et al., 2021). That option of sentiment analysis could thus be transferred to an interactive environment such as the one featured here. We therefore hypothesize that the emotional dimension of responses will gradually change along the experiment’s successive phases, and that said emotional dimension will have a significant relationship with the other dimensions applied to analysis response, which are: (1) the frequency with which each response appears, (2) the number of times each response is copied by participants, and (3) response length as an indicator of response complexity (Orejudo et al., 2022). Moreover, we assume that those three indicators will be interrelated: the model chosen to depict the relationship among variables can well be the path model, as appears below.

### 2.2 Participants

A total of 794 students studying in their first year of bachillerato level (higher secondary education) in Zaragoza (Spain) participated in this experiment. Bachillerato is a non-obligatory level chosen by students who want to continue with university. It consists of two academic years. In the first year, students tend to be 16–17 years old. At the end of the second year, they take the tests that allow them access to university. As our study was anonymous, we did not ask for any age or sex data in order to guarantee maximum anonymity; at any rate, in the Spanish educational system, most of the students who enter bachillerato are 17 years old. In terms of sex, national statistics show very similar percentages. In 2018, 53.1% were girls, and 46.9% were boys (Ministerio de Educación y Formación Profesional, 2020).

To carry out the study, we invited all educational centers in the city of Zaragoza offering bachillerato level to participate in a collective intelligence experiment. The invitation was sent through the Aragon Government Department of Education, i.e., the very entity in charge of organizing education at that level. All centers that agreed to participate under the conditions we proposed (anonymous student participation, student disposal to carry out the task for 2 h and having one computer per student) were included in the experiment. A total of 19 educational centers were allowed access to participate, with students stemming from 33 different class groups. The participating class group with the most students had 33 members, and there were 14 in the smallest.

### 2.3 The task. The ethical dilemma on the *collective learning* platform

The students who participated in this experiment were successfully confronted with two tasks: one consisted of an ethical dilemma, and the other was a mathematical problem. For further details regarding the mathematical task, the reader can consult Orejudo et al. (2022). In the ethical dilemma, lasting about 1 h, the students were asked to analyze a cyberbullying situation: concretely, a sexting case. A girl (Pilar) takes an intimate photo of herself for her boyfriend (Alex), who, in turn, sends it to a male friend (Quino), rising to a challenge. Quino ends up posting it, and the photo thus becomes public. Three questions are asked: (1) What do you think about Pilar taking a photo of herself and sending it to her boyfriend? (2) Should Alex have sent the photo to Quino? (3) Should Quino have posted Pilar’s photo? These questions encourage the participants to answer by formulating open texts without word limits.

### 2.4 Dependent variables

The system recorded each participant’s chosen response to each of the three questions in the course of all seven phases of the experiment. Each participant was allowed to record more than one response per question, one response, or none. A total of 2,897 responses were registered for Question 1, a total of 3,852 answers for Question 2, and 3,864 answers for Question 3. From the participants’ responses, we extrapolated the following variables:

Variables related to the emotional dimension, obtained through the Meaning Cloud program in its demo version^2^:

- Polarity of the analyzed element, expressing an emotional tendency that is either strongly positive (P+, 4 points), positive (2 points), neutral or no polarity (zero points), negative (−2 points), or strongly negative (−4 points)
- Agreement: establishes the agreement among the emotions detected in the text, sentence, or segment to which it refers. It has two possible values: agreement [1 point (Example: “*I think that’s a bad idea*”)] when the different elements have the same polarity, or disagreement (0) when there is no such polarity agreement (“*There is nothing wrong with sending photos of yourself to people you trust. What is problematic is sending photos that could cause you problems to other people. No matter how much you trust that person, you can never be sure that the photo is safe, because your phone could be hacked, stolen or you could end up making a serious mistake because of a silly challenge*”).
- Subjectivity: identifies the subjectivity of the text. It has two possible values: objective (0) (*It’s something she should not have done, although I do not think she’s the one with the problem, as it’s her boyfriend who has shared it without her consent*) when the text has no marks of subjectivity, or subjective when they exist (1) (“*Everyone can do what they think is right, and if they have confidence in it, I do not see it as wrong*”).
- Irony: indicates the presence of irony indicators in the text, with two options: non-ironic or ironic. As can be seen in the results section, the system does not have any detection of ironic responses in the participants’ productions.
- Confidence: represents the degree of confidence associated with the sentiment analysis conducted on the text. Its value is an integer ranging from 0 to 100, according to the system’s score.

Those data are completed by further variables collected by the *Collective Learning* platform:

- Phase: the phase (1, 2, 3, 4, 5, or 6–7) in which each response appeared on the platform (see our above description of phase characteristics in Section 1.3).
- Number of copies: the number of times each response was copied by other participants.
- Final frequency obtained by each response at the end of the experiment (Phases 6–7).
- Length: number of characters of each response. The same indicator has been used in other studies by Orejudo et al. (2022): response length is related to the response’s ethical depth and complexity. This parameter is collected on an Excel spreadsheet by applying the Length formula.

### 2.5 Procedure

Once participating centers had been selected, each center designated a teacher as the local coordinator of the activity. After having accepted to serve as coordinators, those selected teachers attended two training sessions in which they learned about the conditions of the experiment, the way the platform works, and the mode of coordination allowing for all centers to carry out the task synchronously.

Participants’ families were informed through a letter about the study’s purpose and procedure, ensuring participants’ anonymity. In the same letter, the volunteers were informed of their participation and the possibility of excluding from the activity those students whose families did not agree to their involvement or who themselves did not consent to participate. Thus, only students willing to participate in the experiment received the code to access the platform. This study was carried out in accordance with the recommendations of the Council of the British Educational Research Association (BERA) (2011) in the second edition of their Ethical Guidelines for Educational Research. Subjects received no compensation for participating in the study. Compliance with the standards of the Declaration of Helsinki on human experimentation was guaranteed at all times. Furthermore, the entire research program containing this experiment, along with further ones to be carried out in the near future, was approved and validated by the Committee of Ethics in Research of the Government of Aragón (CEICA: Comité de Ética de la Investigación de la Comunidad Autónoma de Aragón).

### 2.6 Data analysis

To compare results among phases, we conducted a one-factor analysis of variance for each quantitative dependent variable, taking the phase as the factor. The two last phases (6 and 7) were analyzed together, given that the system calculated frequencies for those two phases in continuous mode. In Table 1, we provide the values stemming from the Brown-Forsythe robust statistic, as the supposition of equality among variances could not be guaranteed in all cases. For the three dichotomous variables under consideration – Agreement, Subjectivity, and Irony – we calculated the relationship among phases by means of contingency tables, providing the Chi-square Pearson correlation value to contrast the hypothesis of association, and Cramer’s *V* to quantify it.

**Table 1:** Descriptives by phases.

		Question 1			Question 2			Question 3		
		*N*	Mean	DS	*N*	Mean	DS	*N*	Mean	DS
Length	1	922	162.96	100.20	1,446	168.16	100.84	1,053	146.96	93.78
	2	282	223.49	132.74	261	225.13	126.40	249	188.07	104.54
	3	497	229.03	124.96	491	231.73	124.30	517	223.10	125.43
	4	420	249.30	149.69	430	256.25	150.48	378	240.78	136.57
	5	351	248.09	173.55	427	254.40	173.63	1,281	213.67	131.71
	6–7	424	379.46	212.27	784	397.77	207.39	407	227.16	82.39
	Total	2,896	234.73	160.70	3,839	246.51	169.11	3,885	199.25	120.62
		*F* = 115.504, *p* < 0.001, *η*^2^ = 0.185			*F* = 234.015, *p* < 0.001, *η*^2^ = 0.247			*F* = 38.493, *p* < 0.001, *η*^2^ = 0.079		
Number of copies	1	922	1.42	1.36	1,446	1.35	1.38	1,053	1.67	2.39
	2	282	2.19	1.69	261	2.42	1.93	249	2.76	3.86
	3	497	2.28	2.85	491	2.44	3.39	517	2.48	4.26
	4	420	2.25	3.87	430	2.75	5.17	378	2.25	3.97
	5	351	2.93	7.62	427	3.62	9.61	1,281	2.95	7.23
	6–7	424	49.24	47.29	784	53.09	47.11	407	66.06	60.33
	Total	2,896	8.95	24.84	3,839	12.54	29.85	3,885	9.07	28.01
		*F* = 406.908, *p* < 0.001, *η*^2^ = 0.452			*F* = 859.734, *p* < 0.001, *η*^2^ = 0.474			*F* = 442.070, *p* < 0.001, *η*^2^ = 0.485		
Final Frequency	1	922	0.06	0.83	1,446	0.08	0.94	1,041	0.13	1.43
	2	282	0.03	0.38	261	0.11	0.82	248	0.45	2.77
	3	497	0.14	1.58	491	0.25	2.19	517	0.39	2.62
	4	420	0.47	3.16	430	0.92	4.38	377	0.29	2.13
	5	351	0.93	6.63	427	1.58	8.43	1,279	0.79	5.99
	6–7	424	41.71	44.08	784	45.09	44.14	407	56.94	57.84
	Total	2,896	6.33	22.50	3,839	9.55	27.07	3,869	6.39	25.81
		*F* = 364.179, *p* < 0.001, *η*^2^ = 0.425			*F* = 761.068, *p* < 0.001, *η*^2^ = 0.443			*F* = 382.719, *p* < 0.001, *η*^2^ = 0.451		
Confidence	1	922	94.04	5.57	1,054	93.92	5.34	1,053	93.80	5.64
	2	282	92.37	5.72	242	92.26	5.93	249	92.73	5.91
	3	497	92.62	5.82	492	91.46	5.44	515	91.80	5.74
	4	420	92.18	5.75	381	90.89	5.29	376	90.73	5.81
	5	351	92.54	5.97	1,306	91.92	5.63	1,281	92.13	5.87
	6–7	424	90.80	5.44	389	89.27	5.29	407	90.32	5.45
	Total	2,896	92.71	5.78	3,864	92.06	5.65	3,881	92.25	5.86
		*F* = 20.410, *p* < 0.001, *η*^2^ = 0.034			*F* = 46.663, *p* < 0.001, *η*^2^ = 0.060			*F* = 30.957, *p* < 0.001, *η*^2^ = 0.038		
Polarity	1	922	0.13	1.95	1,054	−0.10	1.87	1,053	−0.62	1.95
	2	282	0.21	1.84	242	0.11	1.90	249	−0.76	1.83
	3	497	0.51	1.85	492	−0.19	1.85	517	−0.75	1.89
	4	420	0.35	1.81	381	−0.03	1.91	378	−0.72	1.81
	5	351	0.13	1.83	1,306	−0.26	1.88	1,281	−0.79	1.86
	6–7	424	1.34	1.25	389	−1.57	1.28	407	−1.63	1.20
	Total	2,896	0.41	1.84	3,864	−0.30	1.88	3,885	−0.82	1.85
		*F* = 34.545, *p* < 0.001, *η*^2^ = 0.050			*F* = 49.027, *p* < 0.001, *η*^2^ = 0.055			*F* = 20.80, *p* < 0.001, *η*^2^ = 0.024		

**Figure 1:**
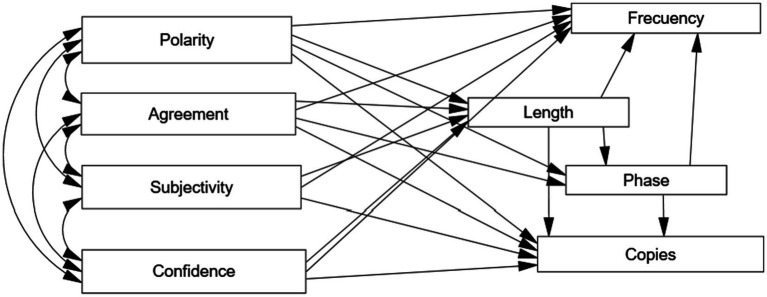
Path diagram.

We then applied path analysis to establish a model of relationships among variables. Path analysis allows the researcher to establish direct relationships among variables and mediating relationships, thereby obtaining an estimation of direct and indirect effects (Byrne, 2010). In this specific case, we considered four exogenous variables related to the responses’ emotional dimensions. We hypothesized that those four exogenous variables, in turn, would partially predict response length. The dimensions of emotions and length, the phase in which each response appeared, and, finally, length and phase would have an impact on the number of copies and the final frequency of responses. We hypothesized those mediating relationships, but also a direct relationship between each exogenous variable, on the one hand, and each endogenous variable and each mediating variable, on the other. The model likewise assumes a relationship between estimation errors of final frequency and the number of copies, given that a relationship between those two variables is expected. The hypothetical model is depicted in Figure 1. As an estimation method, we used ADF through the AMOS 26 statistical program, which provides the ADF option when normal distribution of variables cannot be assumed. Furthermore, we used the goodness-of-fit indicators usually applied in such cases, with their respective cutting-off points (Byrne, 2010).

## 3 Results

As can be observed in Table 1, participant activity in this experiment was not equally distributed along all its successive phases, nor was the response typology always equal. Thus, for the total of three questions, we found a response rate > 1 per participant in Phase 1 (the individual phase) since we had a total of 794 participants. That response rate decreased gradually and notably in Phases 2, 3, 4, and 5 (the phases of interaction with four neighbors: four real-time colleagues whose answers could be viewed by the participant). Then, in Phases 6 and 7, the response rate increases once again (these are the phases in which each participant is allowed to view the group’s most frequent responses). All three questions followed a similar pattern in this respect, although Questions 2 and 3 elicited more responses than Question 1 (see Table 1).

Those differences according to work phase can also be observed in the type of response emitted by the participants. The response length variable increased significantly in the answers appearing in Phases 6–7, and a similar increase can be observed in the transition from Phase 1 (individual work) to Phases 2, 3, 4, and 5 (work in tandem with one’s “neighbors”). The same pattern can be observed in the number of copies made of each response. Responses still present in Phases 6–7 were much more frequently copied than those that appeared in Phase 1, the individual phase. The latter were copied less than the responses appearing in collaborative phases 2, 3, 4, and 5. In terms of final frequency, we only found differences among responses appearing in Phases 6–7 and the remainder (Phases 1, 2, 3, 4, and 5). In all three questions, we observe considerable size effects according to the Eta-square value, and those size effects are associated with the considerable differences that can be noted between Phase 6–7 and the previous ones.

In Table 1, we can compare phases in terms of the program’s own Confidence in its evaluation of responses and the Polarity assigned to them. In the Confidence variable, we find the same pattern as in the ones mentioned above: Phase 1 (individual) generates the greatest degree of Confidence, followed by less Confidence in the 4-neighbor group phases (2, 3, 4, and 5) and even less Confidence in Phases 6–7. Although the response in Phases 6–7 is the one to which the least degree of Confidence can be ascribed according to the system, the mean still lies around 90 points for each of the three responses. Regarding Polarity, we note the following evolution of responses: a notable increment of the positive value from Phase 1 (0.13) to Phase 6–7 (1.34) and an increment of the negative value on Question 2 (−0.15 in the first phase to −1.62 in the last) and of the negative value on Question 3 (−0.62 in the first phase to −1.63 in the last).

The dichotomous variables of Subjectivity and Agreement also evolve notably along successive phases (Table 2). Differences are statistically significant in all three questions. In Phase 1, responses are more objective and have a greater degree of internal agreement, whereas in Phase 6–7 they are more subjective and with a lesser degree of internal agreement. That change occurs progressively in the intermediate phases (2, 3, 4, and 5), where participants work in tandem with their neighbors. Moreover, in Table 2, we can once more observe significant differences among the three questions in terms of these two variables (Subjectivity and Agreement). Ever since the first, individual phase, Question 1 is the one featuring the greatest degree of internal disagreement in the responses (39.7% for Question 1 vs. 33.3% for Question 2 and 34.2% for Question 3). Answers to Question 1 also display a greater degree of subjectivity (74% for Question 1 vs. 54.9% for Question 2 and 48% for Question 3). These differences among questions are maintained during the collaborative phases (2, 3, 4, and 5). However, by the time we reach Phase 6–7, they have become narrower between Questions 1 and 2, while Question 3 remains an outlier (Phase 6–7, Agreement, 72.9% for Question 1, 73.6 for Question 2, and 65.8% for Question 3; Subjectivity, 89.2% for Question 1, 84.2 for Question 2, and 71.0% for Question 3). In terms of irony, no notable differences can be observed, given that the majority of responses lack any sort of irony.

**Table 2:** Agreement, subjectivity, and irony contingency table by phase.

	Agreement								
	Question 1			Question 2			Question 3		
Phase	Disagreement	Agreement	Total	Disagreement	Agreement	Total	Disagreement	Agreement	Total
1	366	556	922	354	700	1,054	360	693	1,053
	39.7%	60.3%		33.6%	66.4%		34.2%	65.8%	
2	169	113	282	119	123	242	120	129	249
	59.9%	40.1%		49.2%	50.8%		48.2%	51.8%	
3	276	221	497	277	215	492	279	236	515
	55.5%	44.5%		56.3%	43.7%		54.2%	45.8%	
4	247	173	420	227	154	381	242	134	376
	58.8%	41.2%		59.6%	40.4%		64.4%	35.6%	
5	193	158	351	685	621	1,306	653	628	1,281
	55.0%	45.0%		52.5%	47.5%		51.0%	49.0%	
6–7	309	115	424	286	103	389	268	139	407
	72.9%	27.1%		73.5%	26.5%		65.8%	34.2%	
Total	1,560	1,336	2,896	1948	1916	3,864	1922	1959	3,881
	53.9%	46.1%		50.4%	49.6%		49.5%	50.5%	
	*χ*^2^ = 145.96, *p* < 0.001 *V* de Cramer = 0.224			*χ*^2^ = 224,425, *p* < 0.001 *V* de Cramer = 0.241			*χ*^2^ = 181.28, *p* < 0.001 *V* de Cramer = 0.216		

Subjectivity									
Phase	Question 1			Question 2			Question 3		
	Subjectivity	Objectivity	Total	Subjectivity	Objectivity	Total	Subjectivity	Objectivity	Total
1	685	237	922	588	466	1,054	505	548	1,053
	74.3%	25.7%		55.8%	44.2%		48.0%	52.0%	
2	221	61	282	177	65	242	141	108	249
	78.4%	21.6%		73.1%	26.9%		56.6%	43.4%	
3	397	100	497	359	133	492	334	181	515
	79.9%	20.1%		73.0%	27.0%		64.9%	35.1%	
4	357	63	420	288	93	381	257	119	376
	85.0%	15.0%		75.6%	24.4%		68.4%	31.6%	
5	275	76	351	922	384	1,306	758	523	1,281
	78.3%	21.7%		70.6%	29.4%		59.2%	40.8%	
6–7	378	46	424	321	68	389	289	118	407
	89.2%	10.8%		82.5%	17.5%		71.0%	29.0%	
Total	2,313	583	2,896	2,655	1,209	3,864	2,284	1,597	3,881
	79.9%	20.1%		68.7%	31.3%		58.9%	41.1%	
	*χ*^2^ = 48.313, *p* < 0.001 *V* de Cramer = 0.129			*χ*^2^ = 133.284, *p* < 0.001 *V* de Cramer = 0.186			*χ*^2^ = 98.67, *p* < 0.001 *V* de Cramer = 0.159		

Irony									
Phase	Question 1			Question 2			Question 3		
	Non-ironic	Ironic	Total	Non-ironic	Ironic	Total	Non-ironic	Ironic	Total
1	921	1	922	1,054	4	1,054	1,052	1	1,053
	99.9%	0.1%		99.6	0.4%		99.9%	0.1%	
2	282	0	282	242	2	242	245	4	249
	100.0%	0.0%		99.2%	0.8%		98.4%	1.6%	
3	496	1	497	492	1	492	510	5	515
	99.8%	0.2%		99.8%	0.2%		99.0%	1.0%	
4	418	2	420	381	2	381	374	2	376
	99.5%	0.5%		99.5%	0.5%		99.5%	0.5%	
5	348	3	351	1,306	18	1,306	1,278	3	1,281
	99.1%	0.9%		89.6%	1.4%		99.8%	0.2%	
6–7	424	0	424	389	0	389	407	0	407
	100.0%	0.0%		100.0%	0.0%		100.0%	0.0%	
Total	2,889	7	2,896	3,864	27	3,864	3,866	15	3,881
	99.8%	0.2%		99.3%	0.7%		99.6%	0.4%	
	*χ*^2^ = 8.851, *p* = 0.115 *V* de Cramer = 0.055			*χ*^2^ = 314.940, *p* = 0.011 *V* de Cramer = 0.062			*χ*^2^ = 19.075, *p* = 0.002 *V* de Cramer = 0.070		

Table 3 displays the estimations of regression weights for the path models generated by each question. In all three cases, we were able to find models that adequately fit the data (P1, *χ*^2^ = 16.494, d.f. = 6; *p* = 0.011; *χ*^2^/DF = 2.749; RMSEA = 0.028; CFI = 0.998; P2: *χ*^2^ = 3.827; d.f. = 4; *p* = 0.430; *χ*^2^/DF = 0.957; RMSEA = 0.000; CFI = 1.00; P3: *χ*^2^ = 3.968, d.f. = 2; *p* = 0.138; *χ*^2^/DF = 1.984; RMSEA = 0.016; CFI = 1.00), excluding regression weights that were not statistically significant (represented by zeros in this table). In all three cases, we obtained a good estimation of response length (41.7, 32.9, and 31.7%). In all three questions, the Confidence variable was the best predictor (*γ*_p1_ = −0.323, *γ*_p2_ = −0.434, *γ*_p3_ = −0.264), followed by Agreement for Question 1 (*γ* = −0.263) and Subjectivity for Questions 2 (*γ* = −0.236) and 3 (*γ* = −0.244).

**Table 3:** Results from path analyses.

			Question 1	Question 2	Question 3
			γ	γ	γ
Length	←	Polarity	0.126	−0.052	−0.1
Length	←	agreement	−0.209	0	−0.172
Length	←	Subjectivity	−0.186	−0.246	−0.245
Length	←	Confidence	−0.320	−0.432	−0.277
Phase	←	Length	0.413	0.242	0.184
Phase	←	Polarity	0.089	−0.121	−0.078
Phase	←	Agreement	0.137	−0.091	−0.085
Phase	←	Confidence	0	0	0
Phase	←	Subjectivity	−0.085	0	0
Number of total copies	←	Length	0	−0.106	−0.044
Number of total copies	←	Phase	0.073	0.349	0.309
Number of total copies	←	Polarity	0.065	−0.189	−0.143
Number of total copies	←	Agreement	0.075	−0.035	0
Number of total copies	←	Subjectivity	0.443	−0.04	−0.063
Number of total copies	←	Confidence	0.287	−0.135	−0.185
Final Frequency	←	Phase	0.267	0.33	0.29
Final Frequency	←	Length	0.449	−0.121	−0.138
Final Frequency	←	Polarity	0.076	−0.177	0
Final Frequency	←	Agreement	0.067	−0.054	−0.034
Final Frequency	←	Subjectivity	0.068	−0.053	−0.061
Final Frequency	←	Confidence	0	−0.130	−0.180
Squared multiple correlations					
Length			0.379	0.331	0.314
Phase			0.173	0.104	0.065
Final Frequency			0.341	0.189	0.156
Number of copies			0.352	0.203	0.170

The prediction of the phase in which the final response appears is more efficacious for Question 1 (28.2%) than for Questions 2 and 3(10.7 and 6.7%, respectively). Among all predictors, Length is the most relevant one (*γ*_p1_ = 0.473, *γ*_p2_ = 0.246, *γ*_p3_ = 0.173). Polarity also appears with a certain relevant weight in all three questions, but with a different sign in Question 1 than in the two others (*γ*_p1_ = 0.123, *γ*_p2_ = −0.110, *γ*_p3_ = −0.084). The two remaining variables, Final Frequency and Number of Copies, are now much better explained for Question 1; moreover, Length and Phase are the two most relevant variables for all questions, as can be observed once again in Table 3. Nevertheless, exogenous variables are also statistically significant, especially Polarity and Confidence in Questions 2 and 3.

The overall relevance of each variable is shown in Table 4, which gathers the total set of effects of each variable within the model. Many of those effects are indirect, i.e., occurring via mediating variables. Thus, Confidence has an indirect effect on Questions 1 and 2 in the Phase variable (*γ*_p1_ = −0.153, *γ*_p2_ = −0.107, *γ*_p3_ = −0.046), on Question 1 in the Final Frequency variable (*γ*_p1_ = −0.219, *γ*_p2_ = 0.021, *γ*_p3_ = −0.015), and on the Number of Copies (*γ*_p1_ = −0.205, *γ*_p2_ = 0.013, *γ*_p3_ = −0.014). We observe similar results for the remaining emotional variables, i.e., Subjectivity, Agreement, and Polarity, with indirect weights, particularly in Question 1.

**Table 4:** Path model total and indirect effects.

	Confidence	Subjectivity	Agreement	Polarity	Length	Phase
	Question 1 – Standardized total effects					
Length	−0.320	−0.186	−0.209	0.126	0.000	0.000
Phase	0.005	−0.077	−0.171	0.141	0.413	0.000
Final frequency	−0.143	−0.036	−0.073	0.170	0.559	0.267
Number of copies	−0.140	−0.031	−0.077	0.172	0.561	0.287
	Question 1 – Standardized indirect effects					
Length	0.000	0.000	0.000	0.000	0.000	0.000
Phase	−0.132	−0.077	−0.086	0.052	0.000	0.000
Final frequency	−0.143	−0.104	−0.139	0.094	0.110	0.000
Number of copies	−0.140	−0.104	−0.142	0.096	0.119	0.000
Question 2 – Standardized total effects						
Length	−0.432	−0.246	0.000	−0.052	0.000	0.000
Phase	−0.104	−0.059	−0.091	−0.134	0.242	0.000
Final frequency	−0.113	−0.043	−0.084	−0.215	−0.041	0.330
Number of total copies	−0.126	−0.035	−0.066	−0.230	−0.021	0.349
	Question 2 – Standardized indirect effects					
Length	0.000	0.000	0.000	0.000	0.000	0.000
Phase	−0.104	−0.059	0.000	−0.012	0.000	0.000
Final frequency	0.018	0.010	−0.030	−0.038	0.080	0.000
Number of total copies	0.009	0.005	−0.032	−0.041	0.084	0.000
	Question 3 – Standardized total effects					
Length	−0.264	−0.244	−0.189	−0.095	0	0
Phase	−0.08	−0.042	−0.104	−0.101	0.173	0
Final frequency	−0.195	−0.065	−0.021	−0.165	0.022	0.287
Number of total copies	−0.197	−0.065	−0.024	−0.170	0.013	0.306
	Question 3 – Standardized indirect effects					
Length	0.000	0.000	0.000	0.000	0.000	0.000
Phase	−0.046	−0.042	−0.033	−0.016	0.000	0.000
Final frequency	−0.015	−0.005	−0.024	−0.026	0.050	0.000
Number of total copies	−0.014	−0.003	−0.024	−0.027	0.053	0.000

## 4 Conclusion

This study’s goal was to analyze the evolution of responses generated by 794 participants who were asked to fulfill the task of analyzing a cyberbullying situation on a Collective Intelligence platform designed to encourage joint collaboration in the elaboration of responses aiming for consensus. The platform’s features are designed to maximize interaction elements that play a key role in the emergence of collective intelligence. as described in our Introduction section.

In the course of the process generated on the platform. The responses’ emotional dimension evolved with clear gradual differences between the initial individual phase (Phase 1). the middle phases of work in small groups (Phases 2, 3, 4, and 5) and the final phases where consensus was sought across the board (Phases 6 and 7). Apart from that relationship with the phases, we found a direct relationship with three further indicators of activity on the platform: (1) length of responses, (2) the final frequency with which certain responses appeared, and (3) the number of times they were copied throughout the system. Moreover, responses evolved differently along time according to the question. The role of the main characters in the ethical case – victim and aggressors – determined that evolution to a large extent.

## 5 Discussion

Regarding these results, it is important to bear in mind that participants’ responses depend on the way the activity on the platform is organized. Phase 1 corresponds with individual work in which participants make their first contribution to the task set before them. In the next four phases (Phases 2, 3, 4, and 5), users share their responses with four “neighbors.” In each of those four phases, the neighbors are different, as well as the conditions under which the responses are displayed (the fixed answers at first, then real-time permutation and permutation of users). This design has the goal of ensuring that interaction among users should take place with a sufficient quantity of information that enables the participants in each group to analyze the responses. It also ensures that responses (thus information) can be distributed throughout the system in its entirety. In this process two aspects are key: the number of participants in each interaction “among neighbors” (five) and the system of permutation of users, designed to rotate user positions and contacts, thus enabling users to go on distributing the information that gradually emerges. This model guarantees interaction, which is key in the emergence of collective intelligence (Navajas et al., 2018). Moreover, the choice to reduce all interaction groups to only five users ensures that each user is confronted with a manageable amount of information. The herd effect is reduced, because the limitation to five neighbors encourages all users to participate actively. As we mentioned in the Introduction section, studies in the field of collective intelligence have found that reduced groups work best (Woolley et al., 2010; Navajas et al., 2018; Toyokawa et al., 2019; Pescetelli et al., 2021). Nevertheless. the design of the *Collective Learning* platform improves upon the model of local work in small groups, given that it manages to spread the information throughout the digital space, ensuring that all participants are allowed to collaborate in seeking the solution for the problem that has been posed: participant neighbor positions rotate and each one of them, in turn, acts as a new node in a series of successive interactions. This strategy optimizes the potential for interaction within the digital space (Almaatouq et al., 2020; Yahosseini and Moussaïd, 2020; Pescetelli et al., 2021).

The results in these small-group phases (Phases 2 to 5) yield similar response patterns for the three questions that made up the task. Compared to Phase 1, the number of responses in Phases 2, 3, 4, and 5 was reduced, thereby implying that the participants were indeed carrying out the task: they were analyzing the other participants’ answers. The fruit of their analysis, in turn, generated new solutions. In the course of Phases 2, 3, 4, and 5, we thus observe that chosen and emergent responses gradually started to lengthen (as shown by the Length variable). Moreover, they gradually tended to be copied more times (i.e., the averages of the number of copies and the final frequency are directly related to the phase in which the responses appear). The emotional polarity of responses also tends to vary, curiously, we ascertain that in Phase 1, polarity is positive; it turns negative in Phases 2 and 3, and also starts to increment. These results might indicate that the platform is achieving the objective we set ourselves: the aim of facilitating an environment that guarantees the spread of information across the entire group. This seems to derive from the direct relationship between the phase in which the response is elaborated and its greater diffusion across the network, observed in the Number of Copies and the Final Frequency, as well as in the fact that the number of neighbors we chose for this experiment (groups of 5) guaranteed a diversity of responses that was sufficient to ensure and encourage response evolution. The conditions we chose also seem to have allowed for an adequate amount of time for the group to develop: the group, as a unit of synchronized analysis, requires such a minimum amount of time to confront ideas and positions through interaction and interchange in order to achieve a maximum degree of cooperation and interdependency in the final consensus. The platform supports and encourages the collective construction of a solution from a perspective of co-responsibility (Palacín and Aiger, 2014; Sierra-Pérez et al., 2021).

Phases 6 and 7 are the phases of consensus and maximum interdependence. To achieve that state, the platform calculates in real time the frequency of each response within the system, and it progressively presents the participants with the “Top 10” answers (the most frequent ones). At the same time, it generates an opposite effect by eliminating the least frequent responses. Users who had chosen less popular answers now have the option of deciding whether they develop a new response of their own pen (Phase 6) or if they decide to adopt one of the Top 10 answers as their own. The mechanism is designed to inspire users to seek consensus: among other methods, it represents an alternative designed to reduce the diversity of responses observed elsewhere and the conditions that lead to such diversity (De Vincenzo et al., 2017; Mann and Helbing, 2017; Massari et al., 2019; Sulik et al., 2022). However, compared to other mechanisms of social influence, this system does not start to function until the responses are elaborated and distributed throughout the network, thereby limiting the initial effect of popularity (Lorenz et al., 2011; Bernstein et al., 2018). The nature of responses is an indication of the importance of structuring the group’s activity by imposing a series of successive phases in time (individual phase, interactive phases, then group phase) in order to encourage the group’s emergent maturity and the synchronicity of its responses, achieved via group consensus. Interaction enhances a user network’s potential for “listening,” for generating discrepancies. For harmonizing points of view, and for strengthening agreements (Palacín and Aiger, 2014; Sierra-Pérez et al., 2021). From an emotional perspective, we observe that the two last phases produce a response pattern that is clearly different from the previous ones. The adopted responses are now even longer and have an elevated degree of final frequency within the system. Of course, the system’s mechanism already tended to encourage such responses; now, however, their emotional dimensions are even more pronounced than in the previous ones; their positive or negative polarity is likewise more extreme than before. In this final phase, the system has fulfilled its purpose: that of reducing the number of responses and proposing a consensus solution. It could be argued. However, that this process has led to a response that is somewhat extreme from an emotional point of view, reflecting a certain “polarization effect” that can easily emerge when small groups interact with the same task (Navajas et al., 2018). However, in a task/situation such as the one proposed herein, the emotional tendency can be viewed as something more positive than negative: it favors a positioning close to the victim’s interests, rejecting those of the aggressors (Machackova and Pfetsch, 2016). It would thus seem that the dynamic generated by the system has exerted a homogenizing effect while producing a response that is formulated with a greater degree of complexity or richness in terms of argumentation. This can be seen in the greater length of favored responses, but also in further indicators stemming from the sentiment analysis: the degree of Confidence in estimations. The responses’ internal agreement among themselves, and the degree of subjectivity.

These indicators, on the whole, present a profile that seems to indicate that a response of greater complexity has been achieved, thereby confirming that a process that aims to generate collective intelligence should produce quality solutions. Moreover, the process itself should also offer a learning experience for those who participate in it (Bernstein et al., 2018; Orejudo et al., 2022). The data resulting from this study gave us a series of clues regarding such complexity from an emotional vantage point. Thus, on the one hand. we have found that the length of responses increases along the successive phases: in the case of all three questions, the path model directly relates that aspect of length with the four emotional dimensions under consideration. Which. taken as a whole, have the capacity to explain a remarkable percentage of responses: 37.9% (for Question 1), 33.1% (for Question 2), and 31.4% (for Question 3).

Moreover, four of the emotional dimensions (except irony) provided by the sentiment analysis tool have significant relationships among themselves and vary in the same manner from one phase to the next. This, in turn, supports our hypothesis that complexity gradually emerges in the course of the system’s successive interaction phases. Emotional valence is increasingly clearly defined as either positive or negative. Two further indicators, Subjectivity and Disagreement, are related, on the one hand, to the responses’ emotional content and, on the other, to the internal structure of the texts that make up those responses. Those three indicators (Emotional Valence, Subjectivity, and Disagreement) are joined by Confidence, which is based on the reliability of the system’s categorization, and which might be associated with less complexity. At any rate, our hypothesis that emotional dimensions can play a relevant role in the interactions that occur on this collective intelligence platform is confirmed by (1) the pattern of responses produced through the experiment, (2) their direct relationship with the Phase variable, (3) the latter’s direct relationship with Length, and (4) the relationship between Length and Final Frequency and Number of Copies. Our data analysis also confirms that the sentiment analysis tool is adequate for the analysis of large quantities of data such as those generated here, or even those generated by interactions on social networks (Karagozoglu and Fabozzi, 2017; Ferchaud et al., 2018; Lozano-Blasco et al., 2021; Zuheros et al., 2023).

The different Polarity valences – positive for Question 1 but negative for Questions 2 and 3 – likewise seem to suggest that these participants have gone through the real process of analyzing the task, as reflected by the successive responses the interaction system has generated. They respond as if they had authentically witnessed the occurrences, achieving a great degree of emotional implication with the actors in the ethical case and also experiencing a certain degree of emotional activation (Caravita et al., 2016). Participants responded by choosing the side of the victim, a choice which generates higher levels of cognitive and emotional empathy with the victim than with the aggressors, as also tends to occur in real-life scenarios (Machackova and Pfetsch, 2016). The group dynamic further accentuated the empathetic response, from a cognitive perspective. Bautista et al. (2022) had already ascertained that the same task and situation tended to improve participants’ moral reasoning capacities. Social neuroscience studies have used group somatic markers (measured by group electrodermal activity) to show that (1) a group needs a certain amount of time to synchronize the act of “listening to one another,” (2) group processes imply improved attention levels in the individual, and (3) emotional activation somewhat attenuates a group’s overall listening capacity (Aiger, 2013). The rate of change tends to accelerate when the group is able to carry out a process of cognitive-emotional revaluation (Vicente et al., 2006) in the phases of cooperation and maximum interdependence (which, in our study, had their equivalent in the final consensus phases. i.e., Phases 6 and 7).

Our results lead us to reflect upon the educational value that might be inherent in contexts of interaction, such as the environment provided by our experiment. Other authors in the field already find a great degree of educational potential in interactive large-group contexts. Large groups allow for their members to construct authentic learning communities; participants can pool and share their resources and they can also create collaborative/cooperative spaces such as the one described herein (Castellanos et al., 2017; Chen, 2018; Ciccone, 2019; Tenorio et al., 2021). Moreover, since such interactive projects take place in “virtual” spaces, the environment is familiar to adolescent users and akin to the type of online space where they are often confronted with threatening risks, such as cyberbullying. Thus, within a virtual space, adolescents can start acquiring the necessary competencies that help them adapt to online environments in general, such as messaging platforms and social networks (Cebollero-Salinas et al., 2022). Such collective intelligence makes it possible to gather a sizeable “crowd” to resolve relevant issues. The system presented in this paper is capable of managing up to 5.000 participants simultaneously in one project. The user can thus feel that they are part of a learning community, or of a collective that takes up joint action when faced with the same problems. This idea of a collective could be a further variable that plays a certain role in the productivity of groups facing a task, such as the group of users featured in this study. An additional advantage found in environments such as this one can be seen in the occasion that it offers for real-time interaction. By adding a fixed time limit, such virtual platforms can provide a consensus mechanism that delivers certain guarantees. We are aware that such group phenomena do not always guarantee success; it is thus essential to describe under which conditions collective intelligence is capable of producing quality solutions (Bigham et al., 2018; Orejudo et al., 2022). The current investigation provides a solid basis for the ongoing study of complexity in collective intelligence. Moreover, certain features of the platform – such as the possibility of modulating the rate of interchange in the small-group work phases, or of choosing the number of responses eliminated in the final consensus rounds – can give rise to different scenarios which, in turn, offer additional alternatives that could be of educational use. If the platform presented herein were programmed to act as a knowledge management system by modifying structures and group processes plus introducing the use of feedback (which, in turn, affects interventions) and by incorporating the aspect of social abilities, it could be reckoned among the leading alternatives in terms of group management designed to efficiently conduct tasks while ensuring that a group can serve as an instrument of change, thanks to the advantages of CI (Collective Intelligence).

However, we should still ask ourselves whether the final consensus phases (Phases 6 and 7) represent the type of interaction that allows users to have a learning experience. From the vantage point of our research, we cannot guarantee it, since social influence seems to be the major factor in these two final stages. But at least the participants are also engaged in social learning, as they are selecting the options they consider best among all the options they had previously been dealing with (Navajas et al., 2018). Other crowd intelligence studies assume that learning is acquired by merely sharing such information, analyzing it and evaluating it (Toyokawa et al., 2014; Hertz et al., 2021); we thus likewise assume that information is being actively processed and that such processing requires a certain amount of previous activity in formulating a response to the question. This, in turn, involves decision-making. Which brings us to the vicinity of active learning theories.

### 5.1 Limitations and prospective

This study has certain limitations. First, we analyzed responses entirely independently of who the authors of those responses were. There thus must have been participants who emitted several responses per phase, contrasted with others who emitted one sole response, or none. The analysis system we employed ascribes the same value to original responses as to those that are copied from other authors in the system. Copied responses are maintained in the system as often as they appear, without applying a process of filtering out duplicates (which would have helped correct the degree of bias). We nevertheless opted to maintain that analysis strategy as it is more ecological: all responses were shared across the network, independently of their origin. Another approach would have consisted in taking each response only once into account, or only to allow one response per participant. It would have entailed that low-frequency responses would have been handled equally in the initial phases. Indeed, in those initial phases, participants seem to familiarize themselves rapidly with the system and seem to be re-recording their own responses on more than one occasion, as is suggested by the high frequency of responses in the individual phase. At any rate, the analysis strategy we chose to apply was in proportion to our research goal, which was to analyze the emotional dimension of responses generated on the platform. At the same time, we were proposing a data analysis based on automatized AI tools that analyze natural language, thereby gathering the entire dynamic generated by the system. Other analysis possibilities admittedly exist, such as evaluating the quality of final responses (Bigham et al., 2018) as a valid option to solve a problem, or studying the degree of creativity that the process gradually produces. In any case, the evolution of AI is creating new possibilities in record time. For now, chatpgt is already emerging as an option to tackle this task efficiently, and may be integrated into platforms such as *Collective Learning* in the near future. Furthermore, we have refrained from analyzing the responses from the vantage point of participants, although that aspect could have improved our grasp of the phenomena under study. For example, we could have analyzed the response patterns that appear in successive phases, or we could have analyzed who were the participants who intervened in the consensus phases, or how the process of social influence acts in a context of entire anonymity such as this one, or which emotional responses are chosen by participants when they interact with others, when they are exposed to the Top 10, or when the system eliminates their responses because the group has had a low degree of acceptance for them. Hertz et al. (2021) and Toyokawa and Gaissmaier (2022) do go more into depth on this level of individual differences: they highlight the importance that such differences can have when responses are displayed to the group and when decisions are taken. Such aspects will need to be taken into account in the future.

## Data availability statement

The raw data supporting the conclusions of this article will be made available by the authors, without undue reservation.

## Ethics statement

The studies involving humans were approved by Research Ethics Committee of the Autonomous Community of Aragon (CEICA). C.P. – C.I. PI22/216. The studies were conducted in accordance with the local legislation and institutional requirements. Written informed consent for participation in this study was provided by the participants’ legal guardians/next of kin.

## Author contributions

SO: Conceptualization, Data curation, Formal analysis, Funding acquisition, Investigation, Methodology, Supervision, Validation, Writing – original draft, Writing – review & editing. RL-B: Conceptualization, Data curation, Formal analysis, Investigation, Methodology, Software, Validation, Visualization, Writing – original draft, Writing – review & editing. PB: Conceptualization, Data curation, Formal analysis, Investigation, Methodology, Resources, Software, Validation, Visualization, Writing – original draft, Writing – review & editing. MA: Conceptualization, Formal analysis, Investigation, Methodology, Supervision, Validation, Visualization, Writing – original draft, Writing – review & editing.

## Footnotes

^1^ https://ic.kampal.com/

^2^ https://www.meaningcloud.com/
